# Pilin Vaccination Stimulates Weak Antibody Responses and Provides No Protection in a C57Bl/6 Murine Model of Acute *Clostridium difficile* Infection

**DOI:** 10.4172/2157-7560.1000321

**Published:** 2016-05-27

**Authors:** Grace A Maldarelli, Hanover Matz, Si Gao, Kevin Chen, Therwa Hamza, Harris G Yfantis, Hanping Feng, Michael S Donnenberg

**Affiliations:** 1 Department of Medicine, Division of Infectious Disease, University of Maryland School of Medicine, Baltimore, Maryland, USA; 2 Department of Microbial Pathogenesis, University of Maryland Dental School, Baltimore, Maryland, USA; 3 Department of Pathology and Laboratory Medicine, VAMHCS, University of Maryland School of Medicine, Baltimore, Maryland, USA

**Keywords:** *Clostridium difficile*, Type IV pilins, Antibody generation, Passive immunization, Mouse model

## Abstract

*Clostridium difficile* is the leading cause of nosocomial infections in the United States, adding billions of dollars per year to health care costs. A vaccine targeted against the bacterium would be extremely beneficial in decreasing the morbidity and mortality caused by *C. difficile*-associated disease; a vaccine directed against a colonization factor would hinder the spread of the bacterium as well as prevent disease. Type IV pili (T4Ps) are extracellular appendages composed of protein monomers called pilins. They are involved in adhesion and colonization in a wide variety of bacteria and archaea, and are putative colonization factors in *C. difficile*. We hypothesized that vaccinating mice with pilins would lead to generation of anti-pilin antibodies, and would protect against *C. difficile* challenge. We found that immunizing C57Bl/6 mice with various pilins, whether combined or as individual proteins, led to low anti-pilin antibody titers and no protection upon *C. difficile* challenge. Passive transfer of anti-pilin antibodies led to high serum anti-pilin IgG titers, but to undetectable fecal anti-pilin IgG titers and did not protect against challenge. The low antibody titers observed in these experiments may be due to the particular strain of mice used. Further experiments, possibly with a different animal model of *C. difficile* infection, are needed to determine if an anti-T4P vaccine would be protective against *C. difficile* infection.

## Introduction

*Clostridium difficile* is a Gram-positive, spore-forming, rod-shaped obligate anaerobe, initially described in 1935 [1]. Currently, it is the leading cause of nosocomial infections in the United States [2,3]. A recent study of nationwide *C. difficile* infection (CDI) morbidity and mortality determined that *C. difficile* was responsible for 453,000 infections and 29,000 deaths in 2011 [4], and recent estimates place excess healthcare costs resulting from CDI in the billions of dollars [2]. Outcomes of colonization with *C. difficile* can range from completely asymptomatic carriage to profuse watery diarrhea, pseudomembranous colitis, toxic megacolon, and death. Disease caused by *C. difficile* is toxin-mediated: the bacterium can secrete two large toxins that target Rho GTPases and induce the massive fluid leakage that leads to the watery diarrhea characteristic of CDI; a third toxin, the *C. difficile* binary toxin, is an ADP-ribosylase that targets Gactin [5] and may assist in bacterial colonization. The most common risk factor for CDI is antibiotic exposure; in a recent meta-analysis of hospital inpatients, antibiotic administration was associated with a 60% increase in risk for CDI [6]. Antibiotic administration leads to disruption of the normal colonic microbiota, which in turn allows *C. difficile* to colonize, proliferate, and cause disease.

Treatment options for symptomatic *C. difficile* include antibiotic therapy with metronidazole, vancomycin, or fidaxomycin. Despite appropriate antibiotic treatment, patients can relapse and disease can recur. Studies place rates of recurrence between 13–50% of first incidence of CDI, and higher if a patient has already had recurrent infection [7,8]. For those who suffer recalcitrant or multiply-relapsing infection, fecal microbiota transplant (FMT) provides another therapeutic option.

Primary prevention, especially in healthcare settings, is critical to preventing morbidity and mortality from CDI. Simple interventions such as handwashing and contact precautions for patients with CDI can decrease spread of the infection. Antibiotic stewardship efforts can also lead to decreased CDI rates; multiple studies have demonstrated that hospital-based interventions designed to decrease antibiotic use overall, and use of antibiotics associated with the development of CDI in particular, have been shown to decrease rates of CDI [9,10].

Another option for primary prevention of CDI is a vaccine directed against *C. difficile*. The *C. difficile* toxins A and B are the most widely-studied vaccine targets, vaccines based on these toxins (fragments or entire protein) have proven successful in preventing signs of CDI in multiple animal models; the antibodies generated by these vaccines have been shown to neutralize *C. difficile* toxins A and B [11,12]. Antibodies against Toxin A correlate inversely with risk of CDI [13]. A recently published phase 1 study of a toxin-based vaccine demonstrated a significant rise in neutralizing anti-toxin antibodies in the individuals administered the experimental vaccine [14]. Other tested vaccine targets include FliC [15], and the cell wall-localized cysteine protease Cwp84 [16]. However, one problem with targeting toxins is that anti-toxin antibodies do not protect against colonization with the bacterium [13], which in turn could lead to its continued spread. In contrast, a vaccine targeting a colonization factor could prevent colonization entirely, which would keep the bacterium from spreading as well as halt the development of clinically apparent disease. Multiple putative colonization factors have been identified in *C. difficile*, including the surface-expressed proteins FliC [15], and Fbp68 [17], the surface-layer protein SlpA [18,19], and type IV pili (T4Ps).

Type IV pili (T4Ps) are thin, hair-like surface appendages widespread in prokaryotes. They have been well characterized in Gram-negative bacteria, including a number of human pathogens such as *Neisseria meningitidis. N. gonorrhoeae, Vibrio cholerae* and other *Vibrio spp., Pseudomonas aeruginosa*, and enteropathogenic *Escherichia coli*. More recently, T4Ps have been described in Gram-positive bacteria as well as in archaeal species [20–22]. The main body of the pilus fiber consists of protein monomers called pilins. The predominant pilin component of the fiber is termed the major pilin; other proteins with similar structures that are incorporated into the pilus at lower frequencies than the major pilin are termed minor pilins. Pilin-like proteins are proteins that have the characteristic sequence or structural features of pilins, but have not been demonstrated to be incorporated into the pilus. T4Ps are involved in colonization, adhesion, motility, and DNA transfer. Pilus fibers can contain multiple different subunits with different roles [23]. Minor pilins can be involved in intracellular adhesion, interaction with host cells, pilus dynamics, and DNA binding [21,23].

As vaccine targets, T4Ps have a number of positive attributes: T4Ps are composed of thousands of repeating monomers, are extracellular and easily accessible to the host immune system; moreover, they are often important for initial colonization and biofilm formation. Vaccines based on T4Ps have proven successful: trials of immunization with T4P subunits or whole pili can confer protection against *V. cholera* [24,25] and *Dichelobacter nodosus* [26], while a Moraxella bovis whole-pilin veterinary vaccine is commercially available (Piliguard^®^ Pinkeye TriView, Merck Animal Health). However, not all T4P-based vaccines have proven efficacious. For example, immunization with *N. gonorrhoeae* PilE was not protective against infection in human trials, despite generating an anti-pilin antibody response [27]. Our previous studies of the immunogenicity and crossreactivity of *C. difficile* pilins demonstrated that they are immunogenic in BALB/c mice [28]; these results led us to hypothesize that immunization with pilins would be protective against infection with *C. difficile*.

Those previous studies also helped us select the pilins included in the vaccine. We demonstrated that immunization with the major pilin, PilA1, led to weak and non-specific responses by ELISA, immunization with the PilJ minor pilin resulted in the generation of strong and specific anti-pilin antibodies, and immunization with PilW, a pilin not yet further characterized, led to broadly reactive anti-pilin antibodies. Indeed, immunization with PilW led to higher anti-PilA1 titers than immunization with PilA1 [28]. PilA1 and PilJ are incorporated into pili and present extracellularly, thus they are accessible to the host immune system [29]. Therefore, we decided to combine PilA1, PilJ, and PilW into an initial pilot vaccine.

Given that *C. difficile* is a colonic pathogen, one part of the pilot experiment involved oral vaccination, to take advantage of mucosal immunity in the same manner as the rotavirus and Sabin polio vaccines and other oral vaccines. The oral vaccine formulation used double-mutant *E. coli* heat-labile toxin (dmLT) as an adjuvant. dmLT has been previously shown to be an effective adjuvant for mucosal vaccines directed against viral as well as bacterial pathogens [30–32]. Another group of mice was vaccinated subcutaneously, using a *Yersinia pestis* lipid A variant as an adjuvant [33]. We hypothesized that immunizations with pilins would result in the formation of anti-pilin antibodies, and that these antibodies would be protective upon challenge with *C. difficile*. The mouse model of acute *C. difficile* infection used here is well established and has been used previously to test other *C. difficile* treatments and vaccines [34,35].

## Materials and Methods

### Pilin expression and purification

PilA1, PilJ, and PilW lacking signal peptides and N-terminal hydrophobic domains were purified as previously described [28]. The N-terminal purification tags were cleaved from each purified pilin protein with recombinant enterokinase (Novagen) and removed by incubation with Ni-NTA resin.

### Vaccine preparation

For the pilot experiment, the vaccine consisted of 100 µg each of PilA1, PilJ, and PilW, and either 25 µg of the adjuvant dmLT, kindly provided by Dr. John Clements [30], for oral immunization or 25 µg of the adjuvant *Y. pestis* lipid A (YPE TBE 44), kindly provided by Dr. Robert Ernst [33], for subcutaneous immunization. Control mice received adjuvant in saline. Vaccines were assembled immediately prior to administration. Each formulation was administered to five mice, for a total of twenty mice used in the entire experiment.

For the first follow-up experiment, mice were immunized subcutaneously with adjuvant plus 100 µg of PilA1, 100 µg PilW, or 100 µg of both proteins. The control group received adjuvant alone. For the second follow-up experiment, mice were immunized subcutaneously with 100 µg PilW or adjuvant alone. For both follow-up experiments, complete Freund’s adjuvant was used for initial immunizations and incomplete Freund’s adjuvant was used for all subsequent immunizations. In both follow-up experiments, each vaccine variant was administered to five mice. Mice in the second follow-up experiment were administered 100 µL of anti-PilW or normal mouse serum by intraperitoneal injection 24 hours prior to challenge. The anti-PilW serum was pooled from five BALB/c mice that were immunized subcutaneously with PilW in a prior experiment and had high titers against PilW, PilA1, PilJ and other pilin proteins [28].

### Animal handling

Five week-old female C57Bl/6 mice (Harlan Laboratory, IN, USA) were maintained in a pathogen-free animal biosafety level 2 facility. All mice used in the experiments were housed in groups of 5 per cage under the same conditions. Food, water, bedding, and cages were autoclaved. For the pilot experiment, mice were immunized three times at ten-day intervals. For the follow-up experiments, mice were immunized four times at ten to fourteen day intervals. Fecal pellets and test bleeds were collected at each immunization. All animals were handled according to Institutional Animal Care and Use Committee (IACUC) guidelines and in accordance with the recommendations in the Guide for the Care and Use of Laboratory Animals of the National Institutes of Health. This study was approved by the University of Maryland Baltimore IACUC as protocol number 0113006.

### *C. difficile* challenge

*C. difficile* challenge was conducted as previously described [34], with minor modifications. Fourteen days after the final immunization, the mice were administered an antibiotic cocktail in drinking water, consisting of 0.4 mg/mL kanamycin, 0.035 mg/mL gentamicin, 850 U/mL colistin, 0.215 mg/mL metronidazole, and 0.045 mg/mL vancomycin, for four days. Two days after the cessation of antibiotics in drinking water, mice were administered an intraperitoneal injection of 10 mg/kg clindamycin. One day after clindamycin administration, mice from the pilot and first follow-up studies were challenged by oral gavage with 10^5^ CFUs of *C. difficile* strain R20291 [36] spores. The mice from the second follow-up experiment were challenged with 10^4^ CFUs of R20291 spores. Mice were euthanized six days after challenge. Fecal pellets and test bleeds were collected on challenge day zero. After euthanasia, necropsy was performed, and terminal bleeds, cecal contents, and colon and cecal tissues were collected and stored at −80°C. Colon and cecal tissue samples were prepared and stained by the University of Maryland Pathology and Histology core facility. Slides were read by a trained pathologist unaffiliated with the lab and scored according to published criteria [37]. The primary endpoint of the studies was the incidence of disease caused by *C. difficile*, defined as the development of diarrhea, loss of 5% of body weight, or death. Secondary endpoints included colonization and histopathology score. To achieve an 80% chance of detecting a difference in incidence of 60% in control animals and 20% in vaccinated animals with a P value less than 0.05, we estimated that 28 animals would be required for each group.

### ELISAs

Unless otherwise noted, all solutions were used at 50 µl/well. Nunc Maxisorp 96-well plates were coated overnight with purified cleaved pilins, brought to 10 µg/mL in phosphate-buffered saline with 0.05% Tween-20 (PBST). Blank wells were coated with plain PBST. After coating, plates were blocked with 5% bovine serum albumin (Sigma) in PBST for 1 hr at 37°C, 100 µL/well. Serum samples diluted 1:500 in PBST were added and serially diluted with one volume PBST in plate. All sera were run in triplicate. For assays testing serum responses, normal mouse serum (KPL) was loaded at 1:500 in PBST. Blank wells were loaded with PBST. Samples were incubated on plate for 2 hours at room temperature. Peroxidase-tagged goat anti-mouse-IgG (H + L) (KPL) was added at a 1:1,000 dilution and incubated for 30 minutes at 37°C. Plates were developed with Sureblue Safestain (KPL) for 30 minutes at room temperature. Optical density at 655 nm (OD655) was read with a microplate reader (BioRad model 680). Blanks were averaged and subtracted from the sample and standard wells. Normal mouse serum (KPL) was used to provide a standard against which the experimental serum could be judged. The average plus two standard deviations of the OD655 with normal mouse serum was taken as the nonspecific normal mouse background OD. For experimental samples, triplicate wells were averaged; the highest dilution with an OD655 greater than normal mouse background was taken as the antibody titer.

ELISAs measuring fecal anti-pilin or anti-dmLT IgA were conducted as described above with the following exceptions. Frozen fecal pellets were re-suspended in 10 µL filter-sterilized PBS per 1 mg fecal mass. Re-suspended pellets were vortexed and centrifuged at 3000×g for 10 minutes to remove debris. The secondary antibody for the fecal IgA ELISAs was horseradish peroxidase-conjugated goat anti-mouse IgA (α) (KPL). Pooled C57Bl/6 mouse fecal pellets from pre-immunization mice were used as the standard background to compare to experimental fecal samples. Wells were coated with dmLT as for pilin.

## Results

### Immunization with a mixture of three pilins leads to low antibody titers in C57Bl/6 mice

**Figure 1:**
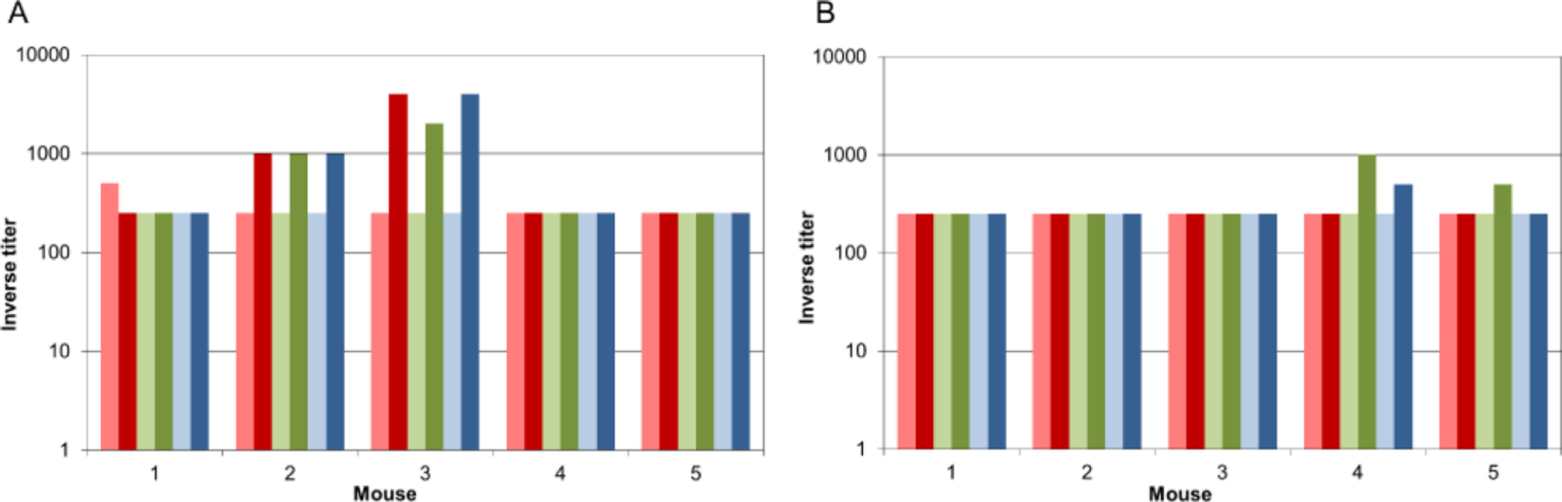
Immunization with a mixture of pilin monomers leads to low anti-pilin antibody titers. Immunizing C57Bl/6 mice with a mix of PilA1, PilJ, and PilW subcutaneously, A) or by oral gavage, B) leads to low anti-pilin IgG and IgA titers respectively. Pale bars show pre-immunization titers, dark bars show pre-challenge test bleed titers. Red bars represent anti-PilA1 titers, green bars represent anti-PilJ titers, and blue bars represent anti-PilW titers.

After three immunizations, mice immunized with pilins demonstrated anti-pilin antibody titers much lower than would be expected, given the results from our previous immunogenicity studies using BALB/c mice [28]. Only two of five mice in the pilin-immunized groups demonstrated anti-pilin antibody titers above background, responding weakly against all three pilins (Figure 1A). In the orally-immunized group, one mouse generated a weak fecal IgA response to all three pilins, whereas one responded weakly only to PilJ (Figure 1B).

**Figure 2:**
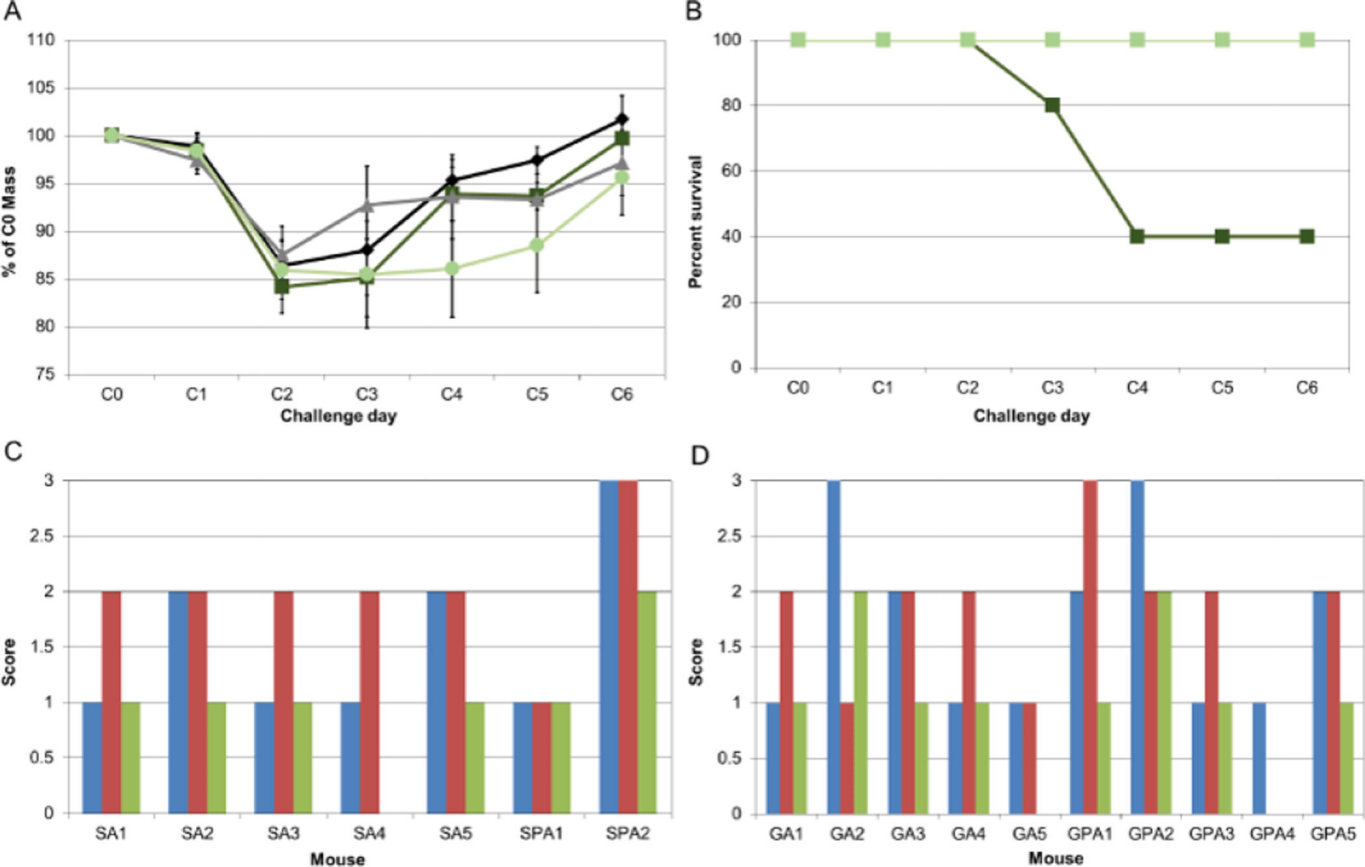
Immunization with a mixture of pilin monomers does not protect against disease caused by *C. difficile*. A) Immunization with pilins confers no protection from weight loss upon infection. Error bars show standard deviation. B) Immunization with pilins affords no protection from mortality. The only mice to die during the experiment were those immunized with pilins subcutaneously. Green indicates mice immunized with pilins and adjuvant. Black/grey indicates mice immunized with adjuvant only. Dark color indicates subcutaneous immunization, pale color indicates gavage immunization. On histological examination, no consistent findings in day 6 cecal pathology are seen in any of the groups, either immunized subcutaneously, C) or by oral gavage. (D) Blue bars show score for neutrophil margination and tissue infiltration. Red bars show score for hemorrhagic congestion and edema of the mucosa. Green bars show score for epithelial cell damage. SPA indicates subcutaneous immunization with pilins and adjuvant. SA indicates subcutaneous immunization with adjuvant alone. GPA indicates gavage immunization with pilins and adjuvant. GA indicates gavage immunization with adjuvant alone. Numbers are individual mice, mice that died prior to challenge day 6 were not evaluated pathologically.

Immunization with a mixture of three pilins did not protect against disease caused by *C. difficile*. All mice lost >10% of body weight by day 3 of infection (Figure 2A). By challenge day 3, three of five mice immunized subcutaneously with pilins had died; none of the other mice in the study died (Figure 2B). No significant difference in weight loss trends was seen among the four different groups. Mice were euthanized on day 6 after infection. Histopathological analysis of colon and cecal tissue harvested after euthanasia demonstrated no difference among the four groups in the three criteria analyzed: neutrophil margination and tissue infiltration, hemorrhagic congestion and edema of the mucosa, and epithelial cell damage (Figures 2C and 2D).

To determine if the low antibody titers in the orally immunized mice were due specifically to poor immunogenicity of the pilins or to a more general failure of the oral vaccination approach, ELISAs were conducted to measure the titers of anti-dmLT IgA in the fecal samples of the control and pilin-immunized mice. ELISAs were performed using the previously described protocol, with wells coated in dmLT. None of the mice in either the control group or the pilin-immunized group demonstrated measurable titers of anti-dmLT IgA, even at low dilutions. A lack of antibodies in both the control and experimental group suggests that dmLT was not an effective adjuvant for oral vaccine delivery in this model.

### PilA1 is not immunosuppressive

**Figure 3:**
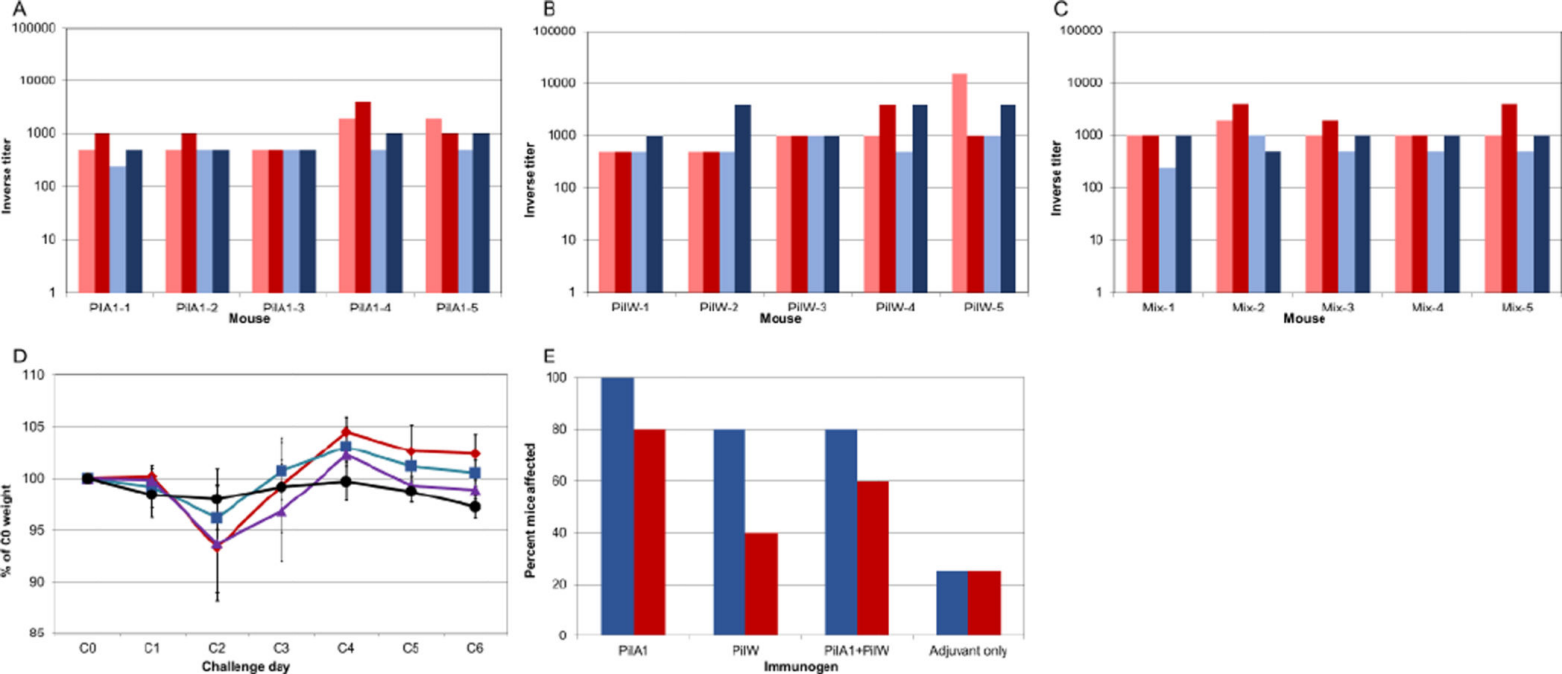
Immunization with PilA1, PilW, or a combination leads to low anti-pilin antibody titers and is not protective upon *C. difficile* challenge. Subcutaneous immunization of C57BL/6 mice with PilA1 A), PilW, B) or both PilA1 and PilW C) using complete/incomplete Freund’s adjuvant generates poor antibody response. Pale bars represent titers after three immunizations; dark bars represent titers after four immunizations. Red bars represent anti-pilA1 titers, blue bars represent anti-PilW titers. D) Immunization with pilins does not protect against weight loss upon *C. difficile* challenge. Red, mice immunized with PilA1; blue, mice immunized with PilW; purple, mice immunized with both PilA1 and PilW; black, mice immunized with adjuvant only. Error bars represent standard deviation. E) Immunization with pilins conferred no protection from *C. difficile* disease upon challenge. Blue bars, percent of mice in each group with diarrhea, loss of >5% body weight on challenge day 2, or both. Red bars, percent of mice in each group with weight loss only.

Because BALB/c mice immunized with PilJ and PilW, but not PilA1, developed high titer antibodies and C57Bl/6 mice immunized with all three proteins developed poor antibody responses, we wished to test the hypothesis that PilA1 is not only poorly immunogenic, but suppresses responses to other antigens. This hypothesis is also supported by the difference in mortality we observed between the immunized and control mice, which suggested that immunization might be detrimental to survival (Figure 3). Therefore, we investigated the possible influence of PilA1 on anti-pilin antibody production. Mice in this experiment were divided into three groups. One group was immunized with PilA1 alone, one with PilW alone, and one with both pilins. A control group received adjuvant alone. Prior experience in BALB/c mice indicated that PilA1 was poorly immunogenic, while PilW elicited cross-reactive responses against all pilins and produced anti-PilA1 responses stronger than those elicited by PilA1 itself [28]. If PilA1 is immunosuppressive, mice immunized with both pilins would have lower anti-pilin antibody titers than mice immunized with PilW alone. We elected to use complete and incomplete Freund’s adjuvant for these rather than YP TBE 44 used in the pilot experiment, because the former was used in the previous immunogenicity studies, where mice produced high-titer anti-pilin antibodies.

Despite an extra booster immunization, C57Bl/6 mice in the follow-up experiments still generated only low-titer anti-pilin antibodies (Figures 3A–3C). However, all five mice in each pilin-immunized group had low antibody titers, as compared to the pilot experiment where only some of the mice showed anti-pilin antibody titers (Figure 1A and Figures 3A–3C). There appears to be no difference in titers among the different groups of mice, despite the different pilins administered as vaccines. All mice in this experiment were challenged with 10^5^ CFUs of *C. difficile*. In contrast to the pilot experiment, all mice survived through challenge day 6. The incidence of signs of disease caused by *C. difficile* in immunized mice was actually higher than in unimmunized mice (Figure 3E). In the groups immunized with PilA1, PilW, and the mix of pilins, five of five, four of five, and four of five mice, respectively, showed some sign of disease caused by *C. difficile*, whereas only one of four mice in the control group showed some sign of disease. We also observed a trend toward less weight loss in the adjuvant-only control group as compared to the groups immunized with pilins, though neither the difference in attack rate nor the weight change was statistically significant. The three groups immunized with pilins did not appear to differ in terms of weight loss (Figure 3D). However, the weight loss in these mice was much less dramatic than that seen in the pilot experiment. This observation could be due to the fact that mice in the follow-up experiment groups were two weeks older than the pilotstudy mice at the time of challenge, due to the extra immunization administered to the mice in the follow-up experiments.

### Systemic passive immunization does not provide detectable intestinal antibodies and is not protective

As immunization of C57Bl/6 mice did not yield antibody titers as high as those previously observed in BALB/c mice, we attempted to determine whether anti-pilin antibodies administered via passive immunization would be protective upon *C. difficile* challenge. Mice in this experiment were immunized with PilW and Freund’s adjuvant, or with adjuvant alone. The mice in the experimental group also received anti-PilW sera generated during previous immunogenicity experiments [28]. Control mice were administered commercial normal mouse serum.

**Figure 4:**
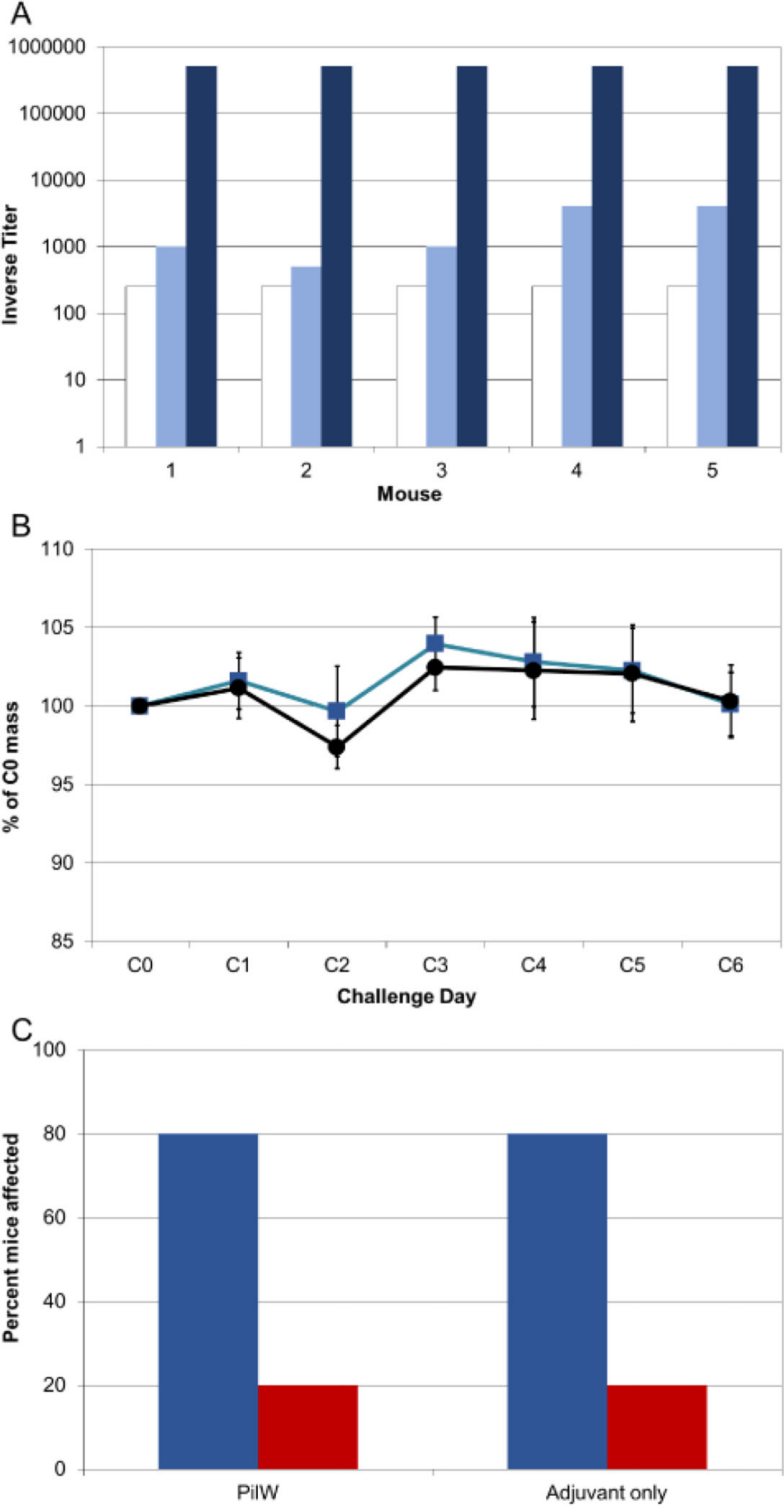
Passive transfer of anti-pilin antibodies leads to high serum anti-pilin antibody titers but does not offer protection against disease caused by *C. difficile*. A) Passive transfer of anti-pilin antibodies leads to significantly higher anti-PilW titers than immunization with PilW. White bars, pre-immunization anti-PilW titers; pale blue bars, anti-PilW titers after four immunizations; dark blue bars, anti-PilW titers one day after passive antibody transfer. B) Passive transfer of anti-pilin antibodies does not protect against weight loss upon challenge with *C. difficile*. C) Passive transfer of anti-pilin antibodies conferred no protection from *C. difficile* disease upon challenge. Blue bars, percent of mice in each group with diarrhea, loss of >5% body weight on challenge day 2, or both. Red bars, percent of mice in each group with weight loss only.

One day after passive transfer of anti-PilW antibodies (i.e. on challenge day 0), all five mice given anti-PilW antibodies had serum anti-PilW antibody titers of 1:512,000 or above (Figure 4A). Mice in the passive-transfer and control group were infected with 10^4^ CFUs; all mice survived to challenge day 6. With this lower challenge dose, only one mouse in each group of five lost >5% of body weight (Figure 4C), and there was no significant difference in weight loss between the two groups. Despite the high serum anti-pilin antibody titers in the passive transfer group, we found the attack rates were equal in the experimental and the control groups (Figure 4D).

To examine whether passively immunized mice had detectable antibodies at the site of infection, IgA and IgG antibodies against PilA1, PilJ, and PilW were measured by ELISA on fecal samples from both the control group and the passively immunized mice. None of the mice in the immunized/passive transfer group exhibited detectable IgA or IgG anti-pilin titers against any of the tested pilins. The lack of anti-pilin IgG suggests that passive immunization was unable to provide protection from *C. difficile* at the site of infection.

Given the results obtained, that the likelihood of observing a statistically significant reduction in the primary endpoint in vaccinated versus control animals if we completed the studies as planned with 28 mice in each group, was virtually nil (38). We therefore elected to terminate the studies.

## Discussion

*C. difficile* is the leading cause of nosocomial diarrhea in the United Stated; a vaccine directed against the pathogen would help alleviate the morbidity and mortality it causes. Vaccines directed against the T4Ps of other organisms have proved successful, and given our previous work on the *C. difficile* pilins, we hypothesized that they would also prove to be good vaccine targets. However, these studies do not support the hypothesis that immunization with pilins confers protection against disease caused by *C. difficile*. There may be several reasons for these results including the suboptimal antibody responses generated, characteristics of the murine model of acute CDI, and the unproven role of T4Ps in infection.

We found that immunization with pilin monomers, whether delivered by an oral or a subcutaneous route, is not effective in generating anti-pilin antibodies in C57Bl/6 mice. The lack of a robust antibody response stands in stark contrast to our experience using BALB/c mice, in which immunization with pilins led to high anti-pilin antibody titers for PilJ, PilU, PilV, and PilW [28]. This difference was not due to choice of route or adjuvant, as we were unable to replicate our results in the C57Bl/6 strain even after reverting to our earlier protocol (Figure 1A, Figure 3A–C, Figure 4A). The two strains of mice have well-recognized differences in immune responses: BALB/c mice have a Th2 bias, whereas C57Bl/6 mice have a Th1 bias [39,40]. This immunological response bias may have been responsible for the poor antibody titers seen in C57Bl/6 mice and in turn suggests that the hypothesis that anti-pilin antibodies are protective against *C. difficile* challenge may not have been adequately tested in these experiments. One solution to this conundrum would be to do immunization and challenge experiments in BALB/c mice; however, *C. difficile* challenge of BALB/c mice appears to result in only mild disease without weight loss, diarrhea or mortality [41].

Along with low titers to pilins, mice orally immunized with dmLT as an adjuvant did not develop antibodies to dmLT. In previous work with that adjuvant, mice immunized with a given antigen and dmLT as adjuvant generated antibodies to both the antigen and the adjuvant [30,32]. We initially wondered whether the lack of antibodies to dmLT could be due to an immunosuppressive effect of PilA1. However, that explanation is unlikely due to the results from our later immunization experiments (Figure 3 and Figure 4). Those subsequent data demonstrated that immunization with individual pilins leads to low anti-pilin antibody titers in the same manner as the mixed-pilin immunization, indicating that the inclusion of PilA1 in the mixed vaccine is unlikely to be the cause of the low anti-pilin antibody titers seen in these studies.

A recent paper examining mucosal immunity in BALB/c and C57Bl/6 mice demonstrated that C57Bl/6 mice produce, at baseline, significantly less fecal and serum IgA than do BALB/c mice. BALB/c mice also have higher titers of innate IgA, that is, IgA with innate recognition of a given pathogen, than do C57Bl6 mice. C57Bl/6 mice were able to mount a pathogen-specific IgA response after infection with invasive Salmonella Typhimurium, but did not do so with a non-invasive mutant, in contrast to the BALB/c mice that generated pathogen-specific antibodies in both cases. From these data, the authors conclude that the generation of specific pathogen-directed IgA virtually requires pathogen bound by innate IgA to be brought to Peyer’s patches, essentially creating a positive feedback loop. BALB/c mice have high titers of innate IgA to initiate this loop, while C57Bl6 generally do not. Since our oral immunization did not involve an invasive bacterium or an interruption of the intestinal epithelium allowing direct access to Peyer’s patches, this process was less likely to start and thus less likely to generate specific anti-pilin or anti-dmLT IgA. Also, given the overall low IgA produced by C57Bl/6 mice and the inferior protective immunity generated by oral immunization in C57Bl/6 mice as compared to BALB/c mice, the former strain may be a suboptimal one as a model for mucosal immunization.

To overcome the poor antibody responses in C57Bl/6 mice, we attempted to administer antibodies passively. Since the *C. difficile* toxins lead to a loss of intestinal barrier function [42], it may be possible to achieve high local intestinal IgG levels early in infection with systemic antibody administration. We found that passive immunization with pooled serum from BALB/c mice immunized with PilW leads to extremely high serum anti-pilin IgG titers, but undetectable fecal anti-pilin IgG (or IgA) titers. Thus, these antibodies do not cross from the circulation into the intestinal lumen, at least at the time that they were measured, and no beneficial effect they may have on response to *C. difficile* challenge was observed. These results cannot rule out the possibility that a mucosal humoral response may be protective against *C. difficile* colonization; however, that hypothesis cannot be addressed with these current data. Given that serum antibodies may not necessarily be transported, mucosal immunization may be a superior option. However, the general characteristics of IgA production in C57Bl/6 mice may make this strain a suboptimal strain in which to test mucosal vaccines.

As C57Bl/6 mice may have suboptimal mucosal vaccine responses due to inherent immunological characteristics of the strain, a clear alternative is to switch to a different model. As mentioned above, BALB/c mice are a poor option for *C. difficile* challenge experiments. Another option is to use a Syrian golden hamster model of CDI; this model has been used to test various vaccines directed against *C. difficile*, including those based on the *C. difficile* toxins and on *C. difficile* FliC [12,15]. Additionally, T4Ps have been observed apparently tethering bacteria to intestinal epithelial cells in hamsters infected with *C. difficile* [43]. The hamster model is a model of acute disease, if T4P are involved in acute infection, immunization and challenge experiments in this model would help demonstrate efficacy of this vaccine.

In preliminary experiments using the same murine acute disease model we used here, our collaborators observed no attenuation of infection with a non-piliated mutant when compared to the parent wild-type strain (Glen Armstrong, personal communication). It should be kept in mind, however, that the C57Bl/6 model of acute CDI requires a cocktail of five antimicrobials followed by administration of clindamycin, which decimates the normal microbiota [44]. If T4Ps are required for *C. difficile* colonization of the colon in the presence of a less-perturbed colonic microbiota, then pathogenesis or immunization studies may be unable to demonstrate an effect using the acute C57Bl/6 model. Alternative approaches to this model include the aforementioned Syrian hamster model, a murine transmission model, a murine long-term colonization model, or a murine relapse model, which use less dramatic pre-exposure antimicrobial regimens [45,46].

In sum, we demonstrate that immunization with *C. difficile* pilin monomers generates only a low titer antibody response in C57Bl/6 mice, a response which is not protective upon challenge with *C. difficile* spores. Passive immunization was also not protective, although anti-pilin IgG was not found in the feces of the passively immunized mice. Further studies in different models and of T4Ps in *C. difficile* are necessary to demonstrate if T4Ps are a viable vaccine target to prevent colonization and infection with the bacterium.
